# Improved filtration performance and antifouling properties of polyethersulfone ultrafiltration membranes by blending with carboxylic acid functionalized polysulfone†

**DOI:** 10.1039/c7ra12447c

**Published:** 2018-02-19

**Authors:** Xing Wu, Zongli Xie, Huanting Wang, Chen Zhao, Derrick Ng, Kaisong Zhang

**Affiliations:** Key Laboratory of Urban Pollutant Conversion, Institute of Urban Environment, Chinese Academy of Sciences Xiamen 361021 China kszhang@iue.ac.cn; University of Chinese Academy of Sciences Beijing 100049 China; CSIRO Manufacturing Private Bag 10 Clayton South Victoria 3169 Australia zongli.xie@csiro.au; Department of Chemical Engineering, Monash University Clayton Victoria 3800 Australia

## Abstract

To improve the filtration performance and antifouling properties of ultrafiltration (UF) membranes, novel polymer blend UF membranes were fabricated in this study. Carboxylic acid functionalized polysulfone (PSFNA) was synthesized by modifying polysulfone (PSF) with 6-hydroxy-2-naphthoic acid (HNA). A series of polymer blend UF membranes were fabricated by adding different amounts of PSFNA into polyethersulfone (PES) to form a homogeneous casting solution. The influences of PSFNA on the morphology, thermal stability, hydrophilicity, filtration performance and antifouling properties of the blend membranes were investigated. The results indicated that by adding PSFNA into PES membranes, the finger-like pores in the membranes became larger, and the porosity and surface hydrophilicity of the membranes were improved. Compared with the pristine PES membrane, PES/PSFNA membranes demonstrated improved filtration performance, resulting in both increased water flux and higher bovine serum albumin (BSA) rejection. At a feed pressure of 0.1 MPa, the PES/PSFNA membrane with 4.0 wt% PSFNA had a pure water flux of 478 L m^−2^ h^−1^, which was 1.7 times higher compared with the PES membrane (287 L m^−2^ h^−1^). In addition, the antifouling properties of PES membranes were also enhanced with the addition of PSFNA. The PES/PSFNA membranes with 3.0 wt% PSFNA had a total fouling ratio (TFR) of 49.6%, as compared with 62.5% for PES membranes.

## Abstract 2

Ultrafiltration membranes with improved filtration performance and antifouling properties have been synthesized through blending polyethersulfone with carboxylic acid functionalized polysulfone.

## Graphical abstract

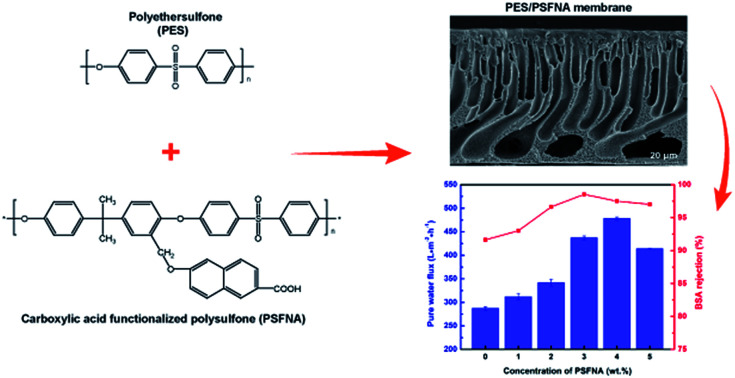

## 1. Introduction

Recently, ultrafiltration (UF) membrane technology has been widely applied in wastewater treatment^1,2^ and drinking water production.^3^ Polymers such as polyethersulfone (PES), polysulfone (PSF) and polyvinylidene fluoride (PVDF) are commonly used as materials to fabricate ultrafiltration membranes.^4,5^ However, due to the natural hydrophobic properties, these polymer membranes are easily fouled. The largest challenge facing the large-scale application of ultrafiltration membranes is membrane fouling, which not only reduces their filtration performance, but also increases the energy consumption during the filtration process.^6–8^

Membrane modification has been reported as one of the most effective approaches to minimize membrane fouling by improving the surface hydrophilicity of the membranes.^9^ Different strategies have been applied to enhance the hydrophilicity of UF membranes. One strategy involves adding hydrophilic nanoparticles such as zirconium dioxide,^10^ zinc oxide,^11^ silver nanoparticles,^12^ tungsten disulfide^13^ or graphene oxide^14^ into the casting solution. Improvement of water permeability was observed in these previous studies. However, this modification method has drawbacks. It was reported that the rejection of UF membranes decreased after embedding tungsten disulfide nanoparticles in membranes.^13,15,16^ Zinc oxide dissolved easily and could release toxic Zn^2+^, which would be harmful for the environment.^13,17,18^ Moreover, the aggregation of nanoparticles also diminished the rejection property of the membranes.^19^

Recently, polymer blending has attracted more attention as a modification method in membrane technology.^20,21^ By blending PES and PSF together into the casting solutions, prepared PES/PSF membranes showed changes in membrane morphology such as pore size, surface roughness and had a higher water permeability.^20,22^ However, the PES/PSF membranes showed lower BSA rejection due to the low compatibility between the two polymers.^20^ Therefore, it was critical to enhance the compatibility between the two polymers, and to reduce the negative effects of segregation of individual polymers.^20^ The introduction of sulfonate into polymers was reported as an effective way to improve compatibility of polymers. Deimede and co-workers found that introducing sulfonate groups into PSF chains increased the compatibility between PSF and polybenzimidazole (PBI).^23^ However, it was reported that adding sulfonated groups into PES/PSF blend membranes reduced the water flux.^20,24^ Compared with sulfonic acid group, carboxyl is a weaker acidic functional group without the swelling phenomenon which has positive effect on improving the polymer compatibility. Performance enhancement has been reported by Liu *et al.*,^21^ in which PSF membrane were modified with carboxylic acid derived from phenolphthalein, which led to improvements in water flux and antifouling properties of membranes. However, to the best knowledge of authors, there is no reported study focusing on the introduction of carboxylic acid functional groups to improve the compatibility in PES/PSF blend membranes.

In this study, a carboxylic acid functionalized PSF (PSFNA) was synthesized by using 6-hydroxy-2-naphthoic acid (HNA) to modify PSF. Novel polymer blend membranes were fabricated by blending PSFNA into the PES casting solution. The objective of this study is to investigate the effect of PSFNA on the morphology, filtration performance and antifouling properties of PES/PSFNA blend UF membranes. The compatibility and thermal stability of PES/PSFNA blend membranes were investigated by differential scanning calorimetry (DSC) and thermal gravimetric analysis (TGA). The effect of PSNFA on the morphology and hydrophilicity of polymer blend membranes were studied by field emission scanning electron microscopy (FESEM), atomic force microscope (AFM) and water contact angle analyses. In addition, the filtration performance and antifouling properties of polymer blend membranes were also investigated by filtrating water flux and bovine serum albumin (BSA).

## 2. Experimental

### 2.1. Materials

Polysulfone (PSF, MW = 35 000 g mol^−1^), chloroform, paraformaldehyde, chlorotrimethylsilane, stannic chloride, polyvinylpyrrolidone (PVP K30) and dimethylformamide (DMF) were purchased from Sigma-Aldrich, Australia. Polyethersulfone (PES, MW = 51 000 g mol^−1^) was purchased from BASF. 6-Hydroxy-2-naphthoic acid (HNA) was bought from ACROS, USA. Triethylamine (TEA) and hydrochloric acid (HCl) were purchased from Merck, Australia. Bovine serum albumin (BSA) was purchased from Amresco, Australia. All the chemicals were analytical grade and used without further purification.

### 2.2. Synthesis of chloromethylated polysulfone (CMPSF)

Chloromethylated polysulfone (CMPSF) was synthesized using the method reported in previous studies.^25^ Briefly, 5 g of polysulfone was dissolved in 250 mL chloroform to form a polysulfone solution. After that, 3.39 g of paraformaldehyde and 12.3 g of trimethylchlorosilane were added into the polysulfone solution in a flask equipped with a reflux condenser and a magnetic stirrer. Then 0.6 g of stannic chloride was added dropwise into the mixture and stirred at 50 °C for 72 h. The prepared solution was added into absolute ethanol and CMPSF was precipitated. After being filtered and washed with ethanol, the precipitated CMPSF was dried in a vacuum oven at room temperature for 12 h.

### 2.3. Synthesis of carboxylic acid modified polysulfone (PSFNA)

**Scheme 1:**
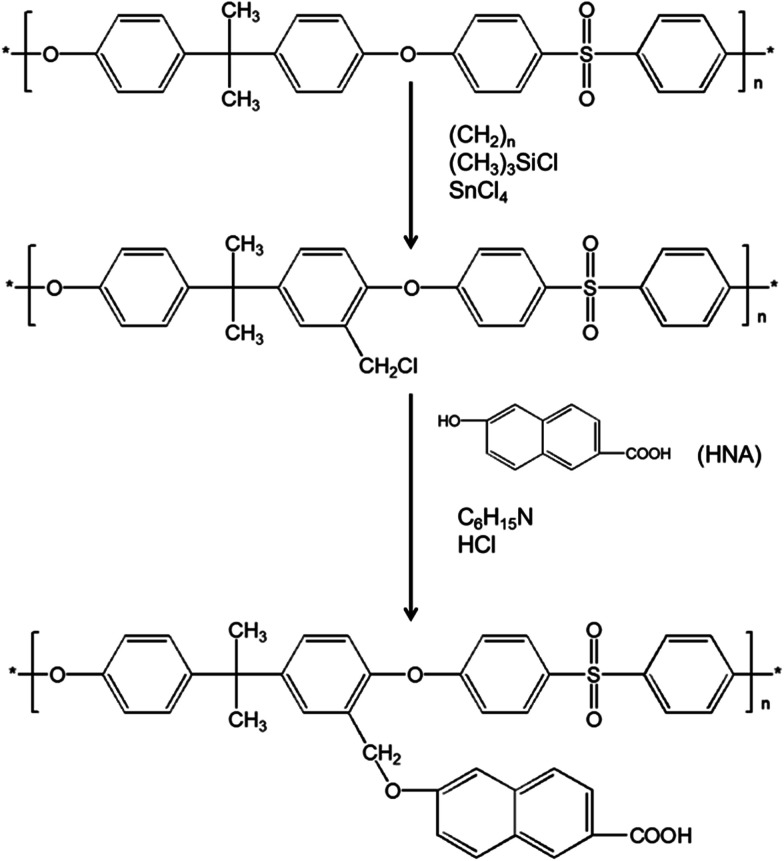
Synthesis of PSFNA.

Carboxylic acid modified PSF (PSFNA) was synthesized by a nucleophilic substitution reaction between 6-hydroxy-2-naphthoic acid (HNA) and chloromethylated polysulfone (CMPSF) as reported in previous studies.^26^ The synthesis scheme of PSFNA is shown in Scheme 1. Typically, 0.5 g of CMPSF was dissolved in 50 mL of DMF in a 250 mL round bottom flask and was stirred by a magnetic stirrer to form a homogenous CMPSF solution. Later, 0.19 g of HNA and 0.45 mL of TEA were added into the CMPSF solution. The reaction was run with a magnetic stirrer at 70 °C for 8 h. After the reaction was completed, 5 mL of 6 mol L^−1^ HCl solution was added into the solution and the resultant polymer was precipitated with ethanol. The resulting PESNA was filtered and washed with ethanol and distilled water, and then dried in vacuum at room temperature for 12 h.

### 2.4. Preparation of ultrafiltration membranes

As shown in Table 1, six types of ultrafiltration membranes were prepared, all of which contained different concentrations of PSFNA. To prepare the casting solutions, PVP, PES and PSFNA powder were mixed in DMF and were stirred at 60 °C to form a homogenous casting solution. After they were well dispersed, the casting solution was kept in an oven at 60 °C overnight to remove air bubbles. Subsequently, the casting solution was dripped on a smooth glass plate and was cast with a casting knife height of 175 μm at ambient temperature. After that, the membrane was immersed in a water bath immediately. Once the membrane was peeled off, it was immersed in another water bath for 24 h to remove residuals. Finally, it was stored in deionized (DI) water till next use. The UF membranes were labeled as M0, M1, M2, M3, M4 and M5 as shown in Table 1. For comparison, the PSF membrane and the PES/PSF-4 membrane containing 4.0 wt% of PSF were prepared with the same method. The casting solutions of PSF and PES/PSF-4 membrane were shown in Table S1.†

**Table 1:** The composition of PES and PES/PSFNA casting solutions

Membrane	PVP (wt%)	PES (wt%)	DMF (wt%)	PSFNA (wt%)
M0	1.5	17.5	81.0	0.0
M1	1.5	16.5	81.0	1.0
M2	1.5	15.5	81.0	2.0
M3	1.5	14.5	81.0	3.0
M4	1.5	13.5	81.0	4.0
M5	1.5	12.5	81.0	5.0

### 2.5. Characterization of membranes

To observe the morphology of the membranes, a field emission scanning electron microscope (FESEM, HITACHI S-4800, Japan) was used to take images of the top surfaces, the bottom surfaces and the cross-sections of prepared membranes. Moreover, membrane surface roughness was analyzed by an atomic force microscope (AFM, Aglient, USA) used in a peak force tapping mode in air.

To investigate the hydrophilicity of the membranes, the water contact angles of the top surfaces of membranes were evaluated by a sessile drop analysis system (CAM200, KSV, Finland). To minimize experimental errors, the average value of contact angles was calculated by randomly selecting five locations on each sample.

Fourier transform infrared spectroscopy (Nicolet 6700 FTIR, Thermo Fisher Scientific Inc., USA) was used to investigate the functional groups on the membrane surfaces. Before analysis, all samples were dried at room temperature for 24 h. An X-ray diffraction study of the prepared membranes was conducted by using a diffractometer (Smartlab, Rigaku, Japan) equipped with a rotating anode Cu-Kα source (45 kV, 200 mA). Data for all samples were collected in the glancing incidence mode at *ω* = 5°, over the 2*θ* range 5° to 90°.

The thermal stability of the membranes was studied by differential scanning calorimetry (DSC, Mettler Toledo DSC-3 system, Mettler Toledeo Corp., Switzerland) and thermal gravimetric analysis (TGA, Mettler Toledo TGA-2 system, Mettler Toledo Corp., Switzerland). For DSC analysis, samples were initially heated from room temperature to 250 °C at a 10 °C min^−1^ heating rate under nitrogen purge gas at 40 mL min^−1^, and then held isothermally for 5 min prior to being cooled to room temperature. For TGA analysis, samples in an alumina crucible were heated from room temperature to 810 °C at a 10 °C min^−1^ heating rate with nitrogen purge at 40 mL min^−1^.

Membrane porosity *ε* (%) of the substrate membranes was measured using a gravimetric method, which was determined by eqn (1):1
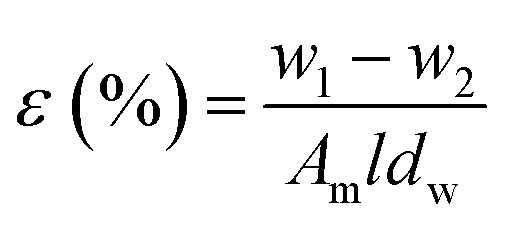
Where *ε* is the porosity of membranes (%), *w*_1_ and *w*_2_ are the weight of wet and dried membrane (g), respectively. *A*_m_ is the membrane area (cm^2^), *l* is the thickness of membranes (cm), and *d*_w_ is the water density (0.998 g cm^3^).

### 2.6. Filtration performance, molecular weight cut-off, and antifouling of membranes

The filtration performance of the membranes was tested in a dead-end filtration system (Sterlitech, HP4750), with an effective area of 14.6 cm^2^. After being pre-compacted at 0.15 MPa for 30 min with the DI water as the feed solution, all membranes were tested at 0.10, 0.15 and 0.20 MPa for 30 min respectively to measure the water flux of the membranes (*J*_0_). The permeate flux (*J*) was calculated with eqn (2):2
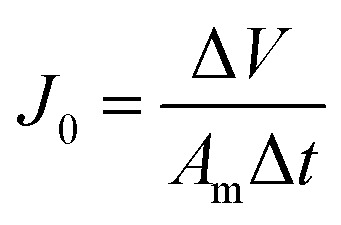
where *J*_0_ is the membrane flux (L m^−2^ h^−1^), Δ*V* is the volume of permeated water (L), *A*_m_ is the membrane area (m^2^), and Δ*t* is the permeation time (h).

Using 1 g L^−1^ BSA solution as the feed solution, the rejection ratio (*R*) of the membranes was tested under 0.1 MPa, and was calculated using eqn (3):3
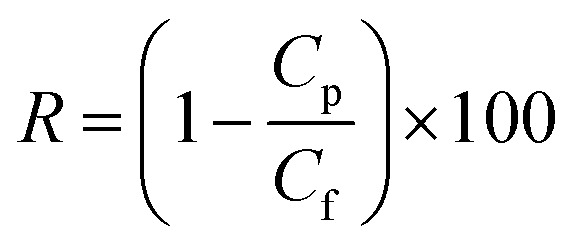
where *C*_p_ and *C*_f_ are the BSA concentrations on the permeate solution and the feed solution respectively (g L^−1^).

The molecular weight cut-off (MWCO) of membranes is represented by the molecular weight of polyethylene glycol (PEG) that is 90% rejected by membranes.^27^ To measure the MWCO of the membranes, the rejections of a series of PEGs with different molecular weights (400, 300, 200, 100, 35 and 20 kDa) were measured. The concentration of PEG was 1 g L^−1^ and the membrane was tested at 0.1 MPa. The concentrations of PEG in the feed solutions and the permeate solution were measured using a total organic carbon analyzer (TOC, TOC-LCSH, Shimadzu, Japan). The PEG rejection was calculated by eqn (3). It was reported that the mean effective pore size was equal to the Stokes radius (*d*_s_) of PEG at 50% rejection, which could be calculated by eqn (4):^27^

4 *d*_s_ = 16.73 × 10^−12^ × *M*^0.557^

New samples of each membrane were used to investigate their antifouling behavior. Firstly, the pure water flux (*J*_0_) of each sample was tested at 0.1 MPa. After that, membranes were used to filter another feed solution which contained 1 g L^−1^ BSA for 4 h. Then the pure water flux (*J*_1_) of the membranes was tested again using the DI water as the feed solution. Later, the fouled membranes were soaked and back-washed by DI water. Afterwards, the pure water flux (*J*_2_) of these membranes was measured again. The antifouling parameters of the membranes were calculated using the following equations:5
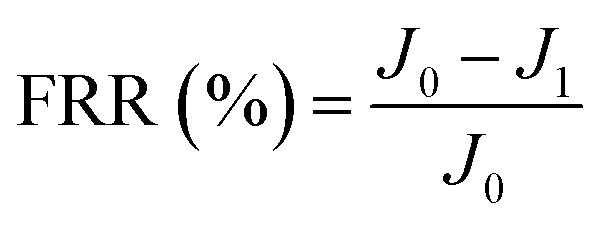
6
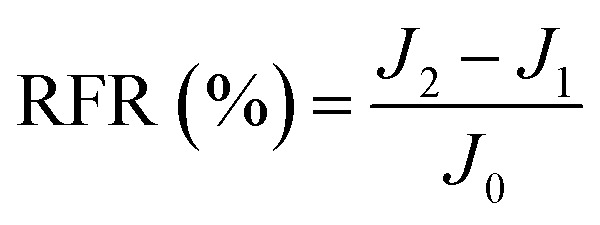
7
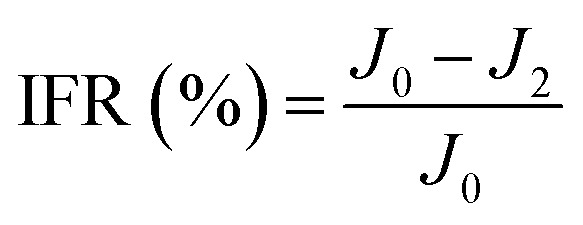
8
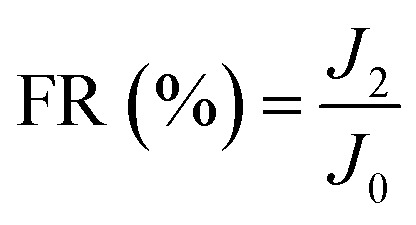
where FRR is the total fouling ratio, RFR is the reversible fouling ratio, IFR is the irreversible fouling ratio and FR is the flux recovery.

## 3. Results and discussion

### 3.1. Morphology

**Fig. 1:**
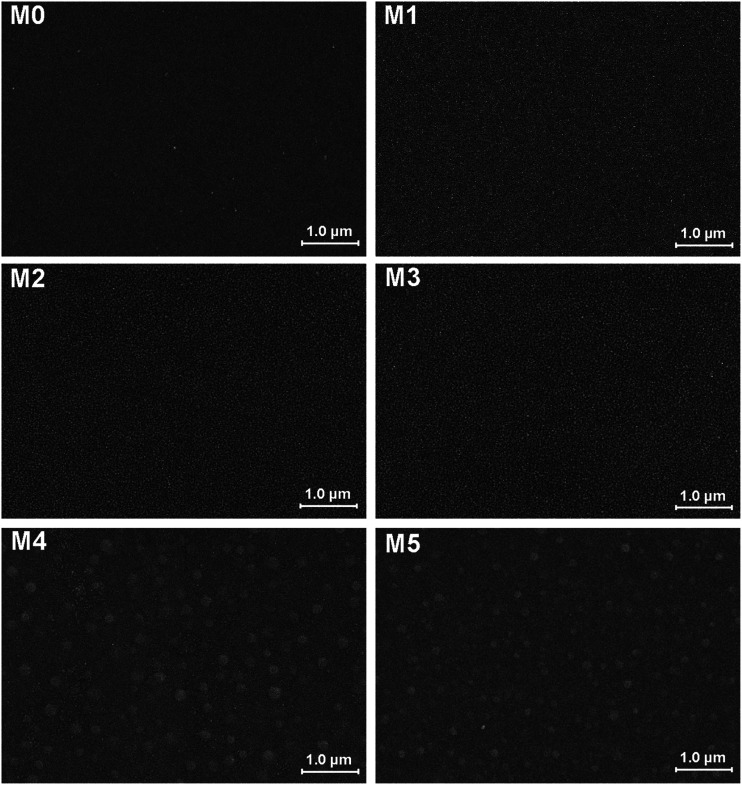
SEM images of the top surfaces of PES/PSFNA membranes.

**Fig. 2:**
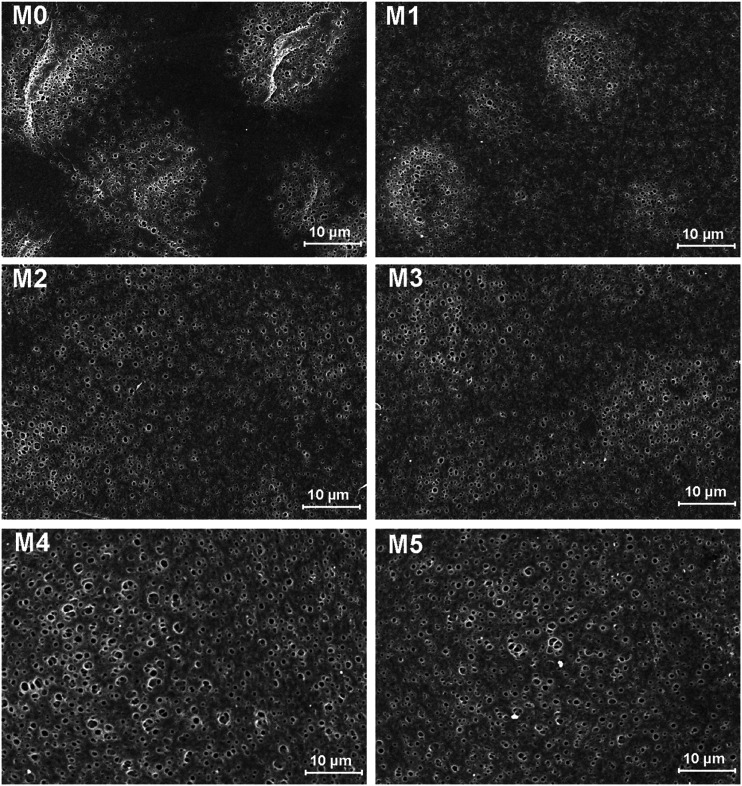
SEM images of the bottom surfaces of PES/PSFNA membranes.

The morphology of prepared UF membranes was studied by SEM. Fig. 1 shows the top surfaces of the PES/PSFNA membranes. The morphology of the top surfaces of the M0 and M1 membranes was almost the same. With increasing concentration of PSFNA, there were more ridge structures on the top surfaces of M2, M3, M4 and M5 membranes. Moreover, compared with M2 and M3 membranes, ridge structures on M4 and M5 membranes were more obvious. Fig. 2 shows the bottom surfaces of membranes containing different amounts of PSFNA. The morphology of the bottom surfaces of membranes containing different amounts of PSFNA were significantly different. It could be observed that on the bottom surface of the M0 membranes, some areas showed pores while some did not. In comparison, when PSFNA was added to the casting solutions, more pores appeared in the M1, M2, M3, M4 and M5 membranes. Using Image J software, the sizes of the pores on the bottom surface of each membrane were measured, and 200 locations were randomly selected on every SEM image to calculate the average pore sizes of the prepared membranes. It could be found that as the concentration of PSFNA in membranes increased, the average pore sizes grew from 0.31 μm in the M0 membrane to 0.37 μm in the M5 membrane. This result indicated that the addition of PSFNA increased the formation of pores on the bottom surface of membranes.

**Fig. 3:**
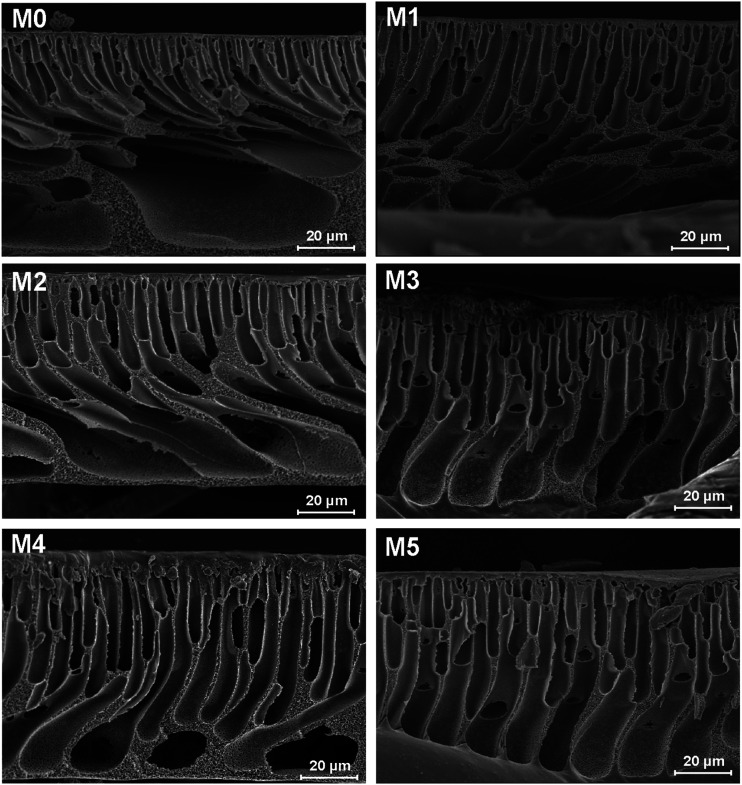
SEM images of cross-sections of PES/PSFNA membranes.

The cross-sections of membranes are shown in Fig. 3. It was found that all membranes had a typical asymmetrical structure, which was composed of a dense skin layer on the top, a finger-like structure in the middle and a macrovoid structure at the bottom.^28^ In the M0 membrane, the finger-like pores were short and oblique, while the macrovoid structures were thick at the bottom. As the concentration of PSFNA increased in the casting solutions, the finger-like pores gradually grew longer, wider and straighter. Moreover, the macrovoid structures became thinner and were gradually replaced by the fully developed finger-like structures as the concentration of PSFNA increased. The larger finger-like structures in PES/PSFNA membranes were caused by the reduced diffusion rate of solvent/non-solvent. Firstly, the added carboxylic groups improved the water-binding capacity of PSFNA, and reduced the diffusion rate of solvent.^21^ Moreover, the viscosity of the casting solution was enhanced by adding PSFNA, which delayed the phase separation process.^28,29^ In addition, the hydrogen bonds between carboxylic groups and PVP retarded the releasing of PVP. As a result of these synergistic effects, the phase separation process lasted longer, meaning that it took more time for the finger-like pores to evolve into the longer and larger structures.^21,28^

**Fig. 4:**
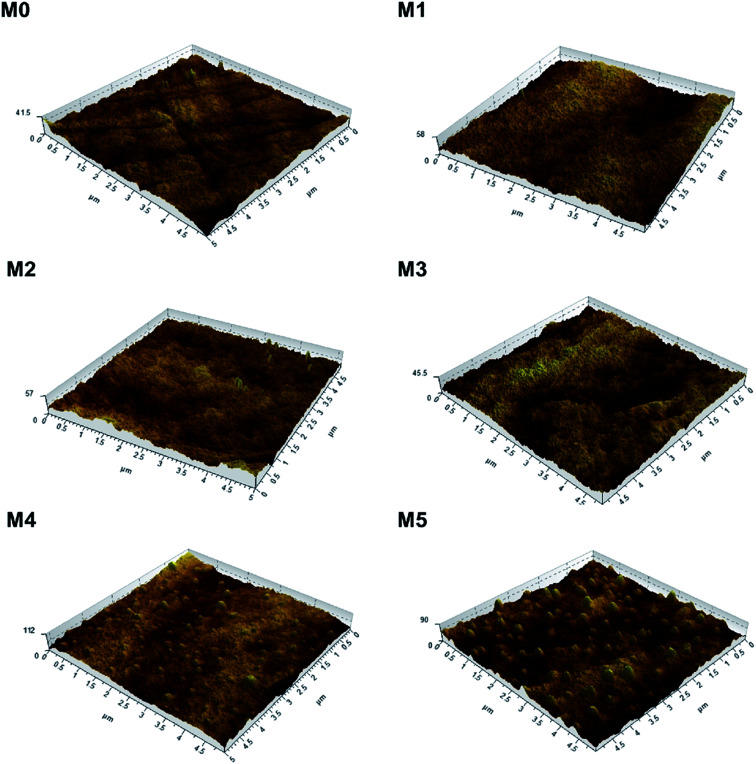
3D AFM images of PES/PSFNA membranes.

To investigate the effect of PSFNA addition on the surface roughness of membranes, AFM analysis was applied. Fig. 4 shows the three dimensional AFM images of top surfaces of the pristine PES membrane and the PSFNA modified PES membranes at a scan size of 5 μm × 5 μm. It could be observed that as the concentration of PSFNA increased in the casting solutions, more ridge structures appeared on the top surfaces of the membranes. Table 2 shows the average arithmetic roughness (*R*_a_), root mean square roughness (*R*_q_) and irregularities (*R*_z_) of prepared membranes. It could be observed that with the addition of PSFNA, membrane surfaces became rougher. As the PSFNA concentration increased from 0 to 3.0 wt%, the *R*_a_ value of membranes increased gradually from 1.36 to 2.08 nm. When the concentration of PSFNA increased to 4.0 and 5.0 wt%, the *R*_a_ value of membranes rose significantly to 5.23 and 7.68 nm respectively.

**Table 2:** Roughness parameters of PES/PSFNA membranes

Membrane	*R*_a_ (nm)	*R*_q_ (nm)	*R*_z_ (nm)
M0	1.36	1.72	9.5
M1	1.60	2.02	10.5
M2	1.88	2.23	11.2
M3	2.08	2.60	14.9
M4	5.23	7.28	36.5
M5	7.68	9.48	37.2

### 3.2. Compatibility and thermal stability

**Fig. 5:**
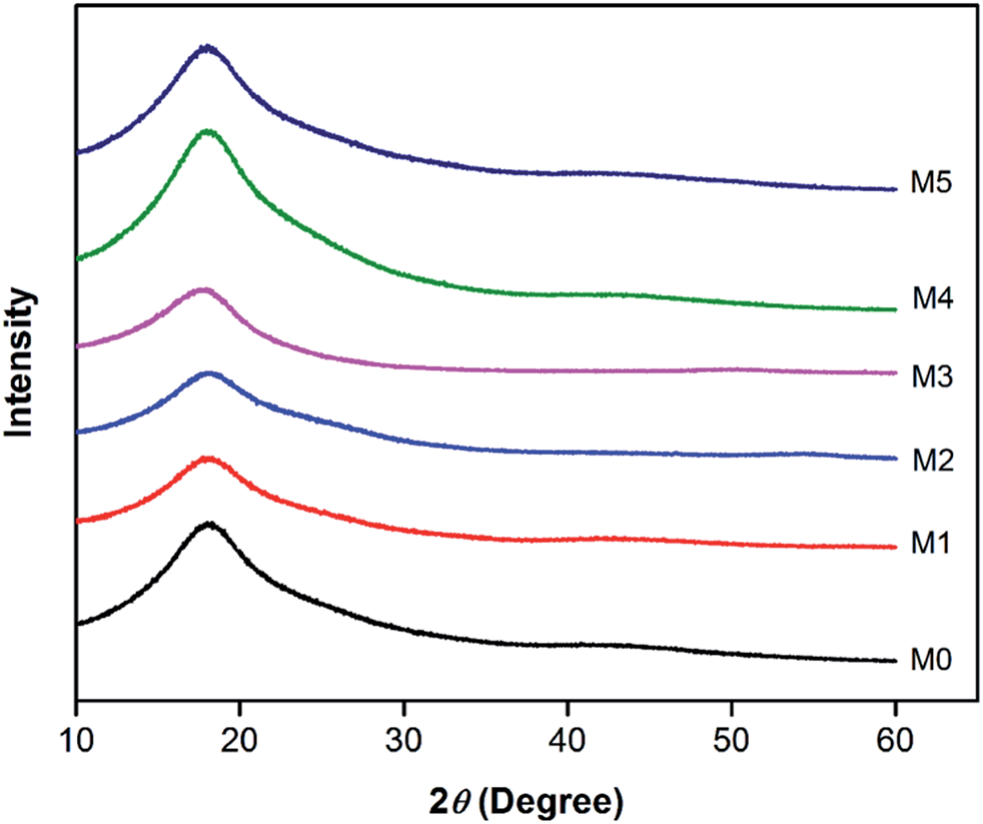
XRD diffraction patterns of PES/PSFNA membranes.

In order to investigate the compatibility between PES and PSFNA, X-ray diffraction patterns of prepared membranes are shown in Fig. 5 from the 2*θ* range of 10° to 60°. It could be observed that all samples showed a single peak at 2*θ* of approximately 18°, which was the typical peak of PES and PSF, indicating they were amorphous in nature.^30–32^ Moreover, all prepared membranes showed almost an identical XRD diffraction pattern, which confirmed the compatibility of PES and PSFNA.^29,33^

**Fig. 6:**
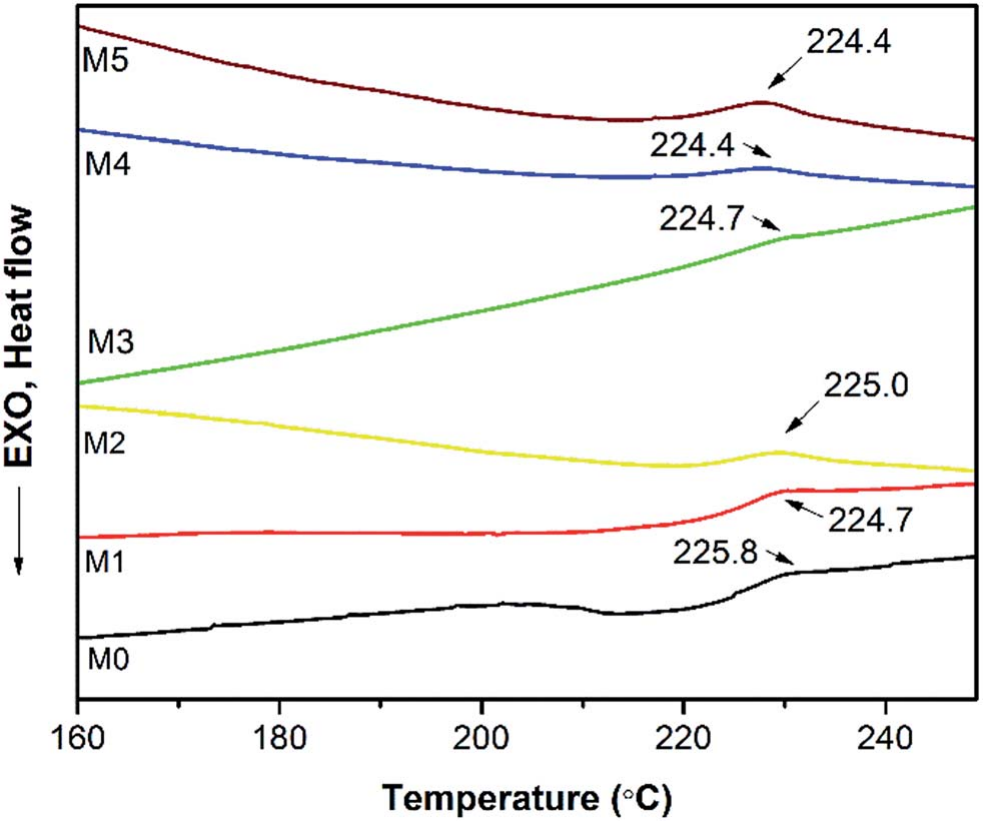
Differential scanning calorimetric (DSC) curves of PES/PSFNA membranes under N_2_ atmosphere.

A differential scanning calorimetric (DSC) measurement of the membranes was applied to investigate whether there are more than one phase in blending polymers.^20,34^ If there is single *T*_g_ in DSC testing, it indicates the polymers are miscible. Fig. 6 presents the DSC curves of prepared PES/PSFNA membranes. It could be found that there was only one glass transition temperature (*T*_g_) in each DSC curve, which indicated that there was no secondary phase transition phenomenon happening in these membranes.^20,29^ For comparison, the DSC curves of the PSF and PES/PSF-4 membranes were shown in Fig. S1.† It could be found that the PES/PSF-4 membrane had two *T*_g_ values, at 188.4 °C and 231.8 °C respectively. The 188.4 °C value is approximate to the *T*_g_ value of the PSF membrane (186.4 °C). This result further verified the compatibility between PES and PSFNA are better than that between PES and PSF.

**Fig. 7:**
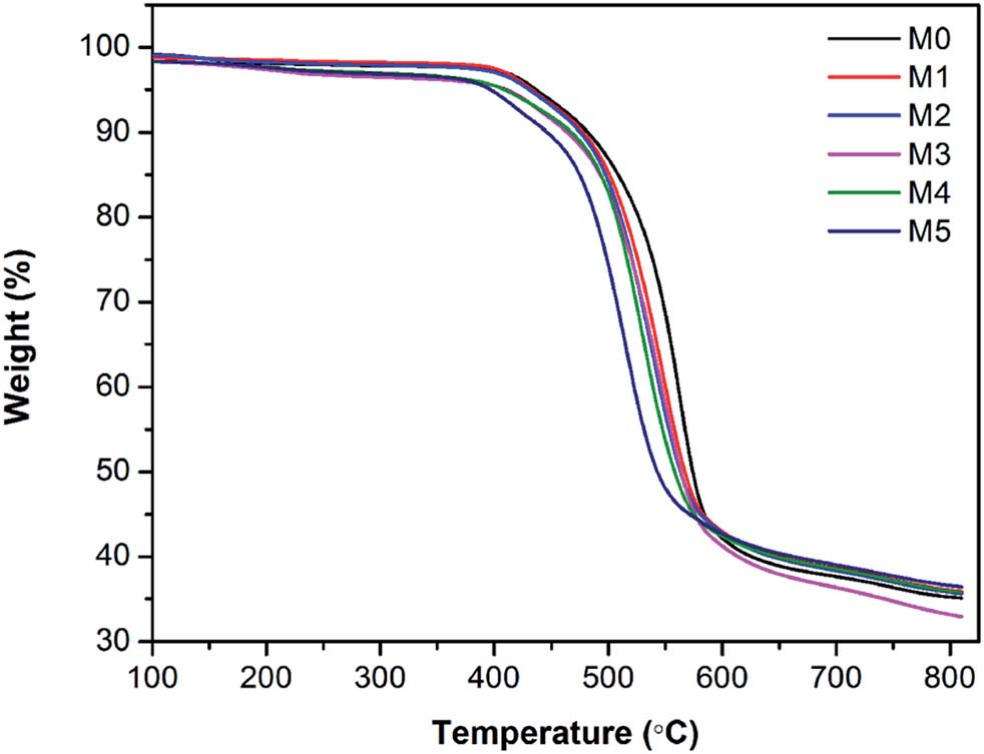
Thermal gravimetric analysis (TGA) of PES/PSFNA membranes under N_2_ atmosphere.

The thermal stability of membranes was further studied by thermal gravimetric analysis (TGA). Fig. 7 shows the TGA curves of PES/PSFNA membranes. It could be observed that all membranes had a similar weight loss profile. Table 3 shows the temperatures at maximum weight loss (*T*_max_) of each membrane. As the concentration of PSFNA increased in the casting solutions, the *T*_max_ decreased from 563.2 °C in the M0 membrane to 510.5 °C in the M5 membrane. This result indicated that the thermal stability of membranes was slightly reduced by adding PSFNA. It was reported in previous studies that the decomposition temperature of carboxylic groups was between 300 to 400 °C.^21,35^ It could be found from Table 3 that the weight loss of membranes between 300 to 400 °C increased as the concentration of PSFNA grew in membranes, which further proved the successful synthesis of PSFNA.

**Table 3:** Thermal performance parameters of PES/PSFNA membranes

Membrane	Weight loss between 300 to 400 °C (%)	*T*_max_ (°C)
M0	0.52	563.2
M1	0.73	547.3
M2	0.84	540.2
M3	1.06	536.7
M4	1.54	527.9
M5	2.18	510.5

### 3.3. FTIR, hydrophilicity and porosity

**Fig. 8:**
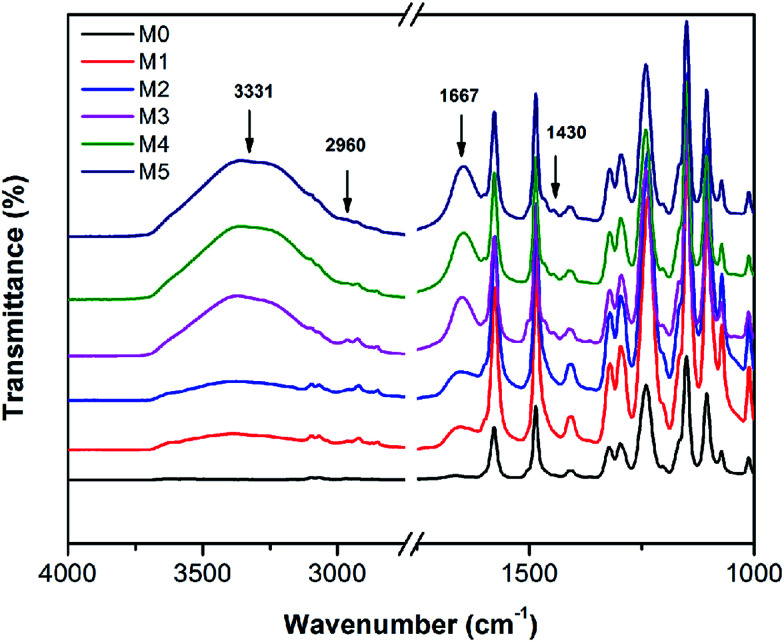
FTIR spectra of PES/PSFNA membranes.

The FTIR spectra of the membranes are shown in Fig. 8. For comparison, the FTIR of the pristine PSF and the PES/PSF-4 membrane were also measured (Fig. S2†). There were similar absorption peaks in all prepared membranes. Peaks at 1322, and 1010 cm^−1^ were associated with asymmetric vibration of O=S=O and asymmetric stretch of C–O.^21^ Peaks at 1415 and 1487 cm^−1^ were due to the vibration of aromatic rings.^21^ Compared with the PES membranes, PES/PSFNA membranes showed a new absorption peak at 2960 cm^−1^ that was attributed to the stretching of the C–H of –CH_3_ in PSF.^21^ In addition, the increased transmittance at 1667 cm^−1^ was due to the C=O stretching vibration of carboxylic groups.^36,37^ New absorption peaks at 3331 and 1430 cm^−1^ could be found in the FTIR spectra of PES/PSFNA membranes, which were due to the stretching vibration of –OH and the asymmetric stretching vibration of COO^−^ in carboxyl groups.^38–40^ Therefore, it could be confirmed that the PESNA was successfully introduced into the PES/PSFNA membranes.

**Fig. 9:**
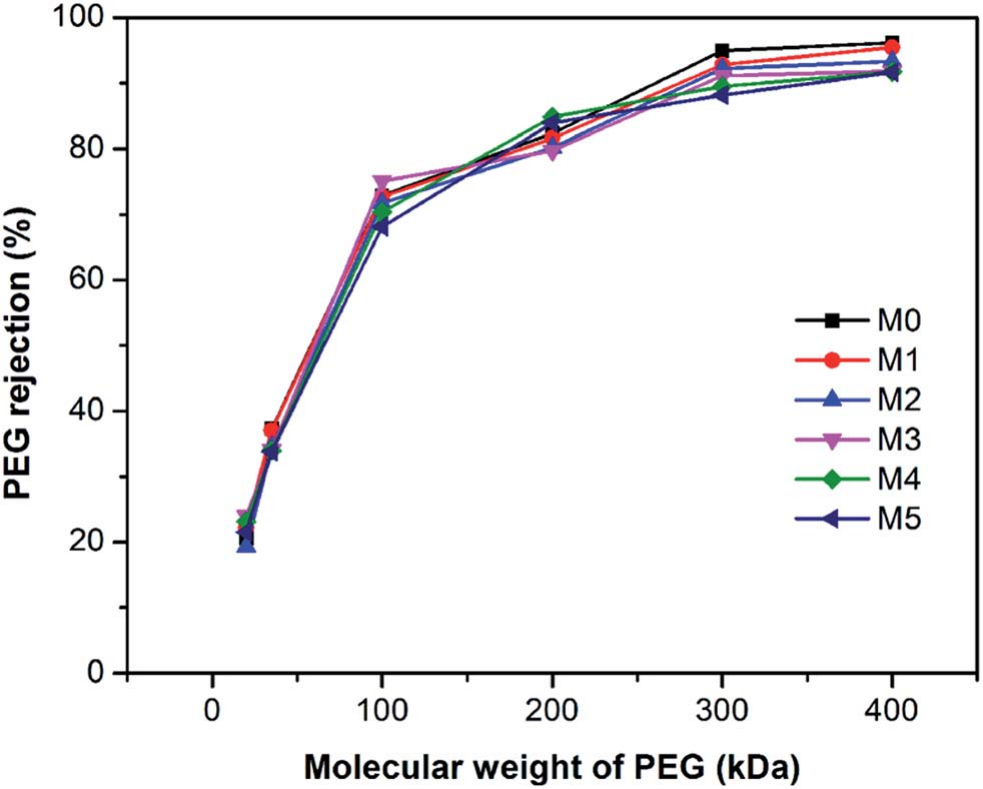
Molecular weight cut-off of PES/PSFNA membranes.

Fig. 9 shows the polyethylene glycol (PEG) rejection of membranes. It could be observed that the molecular weight cut-off (MWCO) of membranes increased with the concentration of PSFNA in the casting solutions. Compared with the pristine PES membranes with 261 kDa of MWCO, the MWCO increased to 276, 285, 290, 326, and 353 kDa for M1–M5 membranes. Based on the PEG rejection data, the mean effective pore sizes of membranes were calculated and demonstrated in Table 4. The results indicated that the effective pore sizes of the membranes increased as the concentration of PSFNA concentrations in the casting solutions was increased.

**Table 4:** Contact angle, porosity and mean effective pore size of prepared membranes

Membrane	Contact angle (°)	Porosity (%)	Mean effective pore size (nm)
M0	85.8 ± 0.87	77.2 ± 0.77	7.6
M1	83.2 ± 0.27	79.5 ± 0.37	7.8
M2	80.5 ± 0.54	82.1 ± 0.37	7.8
M3	78.7 ± 0.50	85.2 ± 0.63	7.8
M4	75.6 ± 0.46	86.6 ± 0.33	7.9
M5	74.0 ± 0.71	85.0 ± 0.63	8.1

Table 4 also shows the contact angles of the top surfaces and the porosity of membranes. It was found that the contact angles of PES/PSFNA membranes were less than those of the pristine PES (M0) membranes, which indicated the improvement of hydrophilicity of PES/PSFNA membranes. In addition, the contact angles gradually decreased as the concentration of PSFNA in the membranes increased from 1.0 wt% to 5.0 wt%. In the phase reversion process, driven by the hydrogen bonds between carboxylic groups and water molecules, the hydrophilic PSFNA potentially moved to the interface between the casting solution and the water bath. As a result, the hydrophilic carboxyl groups existed on the surface of membranes, thus enhancing the surface hydrophilicity.^20,21,29^ The porosity of the membranes gradually increased from 77.2% in the M0 membrane to 86.6% in the M4 membrane, and then slowly decreased to 85.0% in the M5 membrane. The increased porosity was due to the larger finger-like structures and more pores on the bottom surfaces of membranes, which could be observed from SEM images in Fig. 1–3. However, when the PSFNA concentration was increased to 5.0 wt%, the viscosity of the casting solution also increased, which reduced the porosity of the membranes.^19^

### 3.4. Filtration performance

**Fig. 10:**
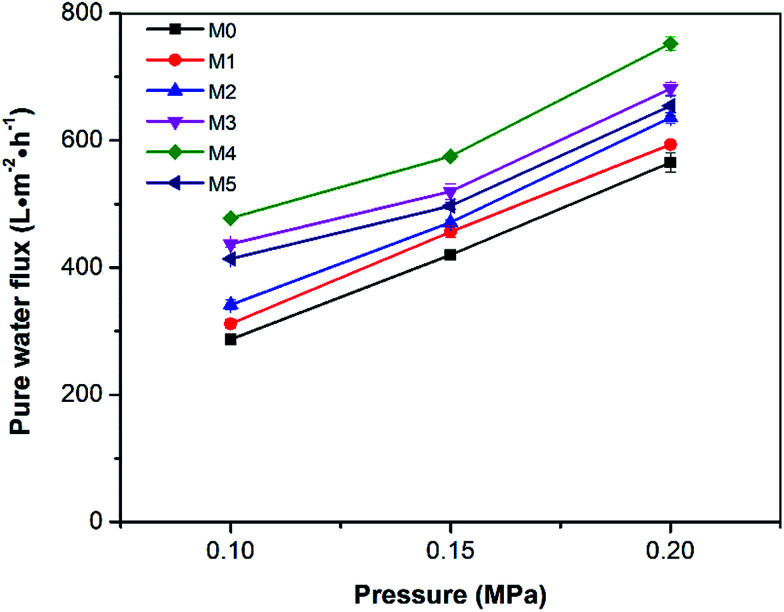
Pure water flux of the membranes under different operating pressures.

The pure water fluxes of the prepared membranes were tested under different filtration pressures (0.1, 0.15 and 0.20 MPa) and the result are shown in Fig. 10. It can be seen that the water flux of the membranes increased with increasing filtration pressure, which could be attributed the larger driving force provided by higher pressures. Although the water flux of PES/PSFNA varied with the concentration of PSFNA, compared to the pristine PES membrane, PSFNA modified membranes had higher water flux at all of the three operating pressures. This was probably due to the combination of the improved hydrophilicity, the increased porosity and surface roughness of PSFNA modified membranes. Moreover, it is interesting to observe that the water flux correlated with the porosity of the membranes. When the PSFNA concentration increased from 0 to 4.0 wt% in membranes, the water flux showed an upward trend from 287 to 478 L m^−2^ h^−1^ at 0.1 MPa, and then declined to 413 L m^−2^ h^−1^ when the membrane contained 5.0% of PSFNA. When compared with the M5 membrane, the porosity of the M4 membrane was higher but the mean effective pore size was lower. This result indicates that the increased porosity is the dominant factor in improving the water flux of the membranes prepared in this study.

**Fig. 11:**
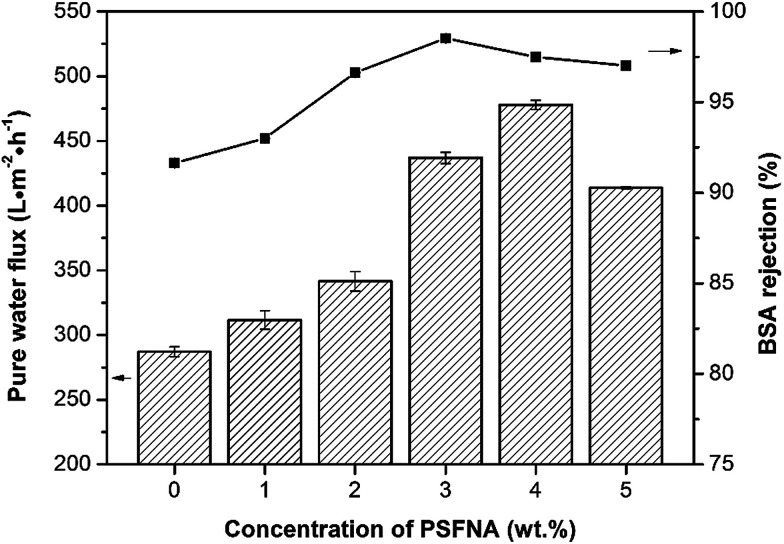
Water flux and BSA rejection of prepared membranes with different concentration of PSFNA. 1 g L^−1^ BSA solution was selected as the feed solution, and the filtration pressure was 0.1 MPa.

The BSA rejection performance of prepared membranes was measured by filtering 1 g L^−1^ BSA solution. It was found that compared to the pristine PES membrane, PSFNA modified membranes showed higher BSA rejection (Fig. 11), which was likely due to the following two reasons. Firstly, the surfaces of the PES/PSFNA membranes were more hydrophilic because of the existence of carboxyl groups. Because of the interactions between carboxyl groups and water molecules, the water molecules were easily attached to the surfaces of the membranes and formed a thin hydration layer between the foulants and the membrane surfaces.^21^ This hydration layer could not only increase the permeability of the membranes, but could also impede the contact between BSA and the membrane surfaces.^20,21^ Secondly, BSA is negatively charged at pH 7.4,^41^ while PES/PSFNA is also negatively charged because of the existence of carboxyl groups.^42^ The electrostatic repulsion between both negatively charged membrane surfaces and BSA also impeded the attachment of BSA to the membrane surface. As a result, the BSA rejection of PES/PSFNA membranes increased.^42,43^ However, due to the increasing pore sizes, the BSA rejection in the M4 and M5 membranes was lower than that in the M3 membrane. To investigate the influence of the addition of carboxylic groups on membrane performance, the water flux and BSA rejection of the PES/PSF-4 membrane were measured to compare with the M4 membrane (Table S2†). It was found that the water flux in the PES/PSF-4 membrane was 817 L m^−2^ h^−1^ under 0.1 MPa, while the BSA rejection was merely 43.8%. This result indicated that the high water flux of the PES/PSF-4 membrane was due to the defect on the membrane caused by self-aggregation of PES and PSF, corresponding to the DSC result and previous studies.^20^

### 3.5. Antifouling performance of membranes

To investigate the antifouling property of the membranes, the fouling parameters TFR, RFR, IFR and FR were measured. The TFR is defined as the water flux reduction due to membrane fouling.^29^ Larger TFR values indicated more serious membrane fouling. It was found that compared with PES and PES/PSF-4 membranes, PES/PSFNA membranes showed smaller TFR values (Table 5). This result indicates that PES/PSFNA membranes had better antifouling performance than the pristine PES membranes. RFR and IFR are degrees of water flux reduction caused by reversible fouling and irreversible fouling of membranes. Therefore, a higher ratio of RFR to TFR of a membrane indicates that more fouling could be removed by a physical cleaning process. In the pristine PES membrane, the ratio between RFR to TFR was 42.1%, while the ratio increased to 58.7% for the M3 membrane. In addition, it was also found that the flux recovery (FR) values of PES/PSFNA membranes were higher than that of the pristine PES membrane, which indicated that the degree of water flux recovery of membranes was improved by modifying them with PSFNA. Moreover compared with the M4 membrane, when the PSF at the same concentration was applied to modify the PES membrane, the PES/PSF-4 membrane showed larger TFR and lower FR. In addition, the ratio between RFR to TFR of the PES/PSF-4 membrane was only 34.1%. This result indicated that after adding hydrophilic carboxylic groups, the PES/PSFNA membranes showed better fouling resistance than the PES/PSF-4 membrane. As shown in Tables S2† and 4, the contact angle of the PES/PSF-4 membrane was 84.5° ± 0.92°, while the contact angle of the M4 membrane was 75.6° ± 0.46°. In addition, compared with the pristine M0 membrane, the PES/PSFNA membranes also showed better antifouling property. The potential reasons for the enhanced antifouling performance of PES/PSFNA membranes are the improved surface hydrophilicity and the electrostatic repulsion between the membranes and BSA, which reduced the attachment of BSA on the membrane surfaces despite the surface roughness of membranes increasing slightly.^44^ However, as the concentration of PSFNA increased in the casting solution, the surface roughness of M4 and M5 membranes rose significantly. A rough membrane surface had more valley structures, which allowed foulants to attach easily, and consequently reduced the antifouling property of membranes. As a result of the opposing effects of improved surface hydrophilicity and rougher surfaces, the FR values of the M4 and M5 membranes were lower than that of the M3 membrane.

**Table 5:** Fouling parameters of prepared membranes

Membrane	TFR (%)	RFR (%)	IFR (%)	FR (%)
M0	62.5 ± 0.6	26.3 ± 1.0	36.2 ± 0.7	63.8 ± 0.7
M1	59.8 ± 0.8	24.6 ± 0.7	35.2 ± 0.6	64.8 ± 0.6
M2	54.6 ± 0.6	26.5 ± 1.0	28.1 ± 0.8	71.9 ± 0.8
M3	49.6 ± 0.4	29.1 ± 0.1	20.5 ± 0.3	79.4 ± 0.3
M4	50.2 ± 0.2	26.1 ± 0.7	24.1 ± 0.6	75.9 ± 0.6
M5	50.9 ± 0.5	21.5 ± 0.9	29.4 ± 0.8	70.6 ± 0.8
PES/PSF-4	64.8 ± 1.7	22.1 ± 1.3	42.7 ± 2.6	57.3 ± 2.6

## 4. Conclusion

In this study, carboxylic acid functionalized PSF was synthesized by functionalizing PSF with HNA. The influences of PSFNA on the morphology, filtration performance and antifouling properties of PES membranes were investigated. It was found that modifying PSFNA with PES, the morphology of the membranes was changed. Compared with the PES membranes, PES/PSFNA membranes had larger finger-like structures. The membrane porosity also increased from 77.2% to 86.6% when 5.0 wt% PSFNA was added to the casting solution. Moreover, the hydrophilicity of the PES/PSFNA membranes was also improved. Despite an increased surface roughness, which had a negative effect, these changes enhanced the filtration performance of PES/PSFNA membranes by improving the water flux and BSA rejection. Compared with PES membranes, the BSA rejection of the PES/PSFNA membranes with 4.0 wt% PSFNA increased from 91.6% to 97.5%, while the pure water flux was increased 1.7 times, from 287 to 478 L m^−2^ h^−1^ at a feed pressure of 0.1 MPa. In addition, the PES/PSFNA membranes had lower TFR and higher FR, which indicated that the antifouling properties of PES membranes were also enhanced by the addition of PSFNA.

## Conflicts of interest

There are no conflicts to declare.

## Supplementary Material

RA-008-C7RA12447C-s001
