# Supplementary material for: Improved filtration performance and antifouling properties of polyethersulfone ultrafiltration membranes by blending with carboxylic acid functionalized polysulfone
Source: RSC Adv. 2018 Feb 19;8(14):7774–84. doi: 10.1039/c7ra12447c (PMC9078463; doi:10.1039/c7ra12447c)
Supplement: RA-008-C7RA12447C-s001 [file RA-008-C7RA12447C-s001.pdf]

Electronic Supplementary Material (ESI) for RSC Advances.
This journal is © The Royal Society of Chemistry 2018

Supplementary Information

Improved filtration performance and antifouling property of polyethersulfone ultrafiltration membranes by blending with carboxylic acid functionalized polysulfone

Xing Wu[a,b,c], Zongli Xie*[c], Huanting Wang[d], Chen Zhao[d], Derrick Ng[c], Kaisong Zhang*[a,b]

1. *Key Laboratory of Urban Pollutant Conversion, Institute of Urban Environment, Chinese Academy of Sciences, Xiamen, 361021, China. Email: kszhang@iue.ac.cn;*
2. *University of Chinese Academy of Sciences, Beijing, 100049, China*
3. *CSIRO Manufacturing, Private bag 10, Clayton South, Victoria 3169, Australia. Email: zongli.xie@csiro.au*
4. *Department of Chemical Engineering, Monash University, Clayton, Victoria 3800, Australia*

Figure Captions

Table S1. The composition of PSF and PES/PSF-4 casting solutions.

Fig. S1. Differential scanning calorimetric (DSC) curves of PSF and PES/PSF-4 membranes under N_2 atmosphere.

Fig. S2. FTIR spectra of the PSF membrane and PES/PSF-4 membrane.

Table S2. Pure water flux, BSA rejection and contact angle of the PES/PSF-4 membrane.

Table S1. The composition of PSF and PES/PSF-4 casting solutions.

Membrane	PVP (wt.%)	PES (wt.%)	DMF (wt.%)	PSF (wt.%)
PSF	1.5	0.0	81.0	17.5
PES/PSF-4	1.5	13.5	81.0	4.0

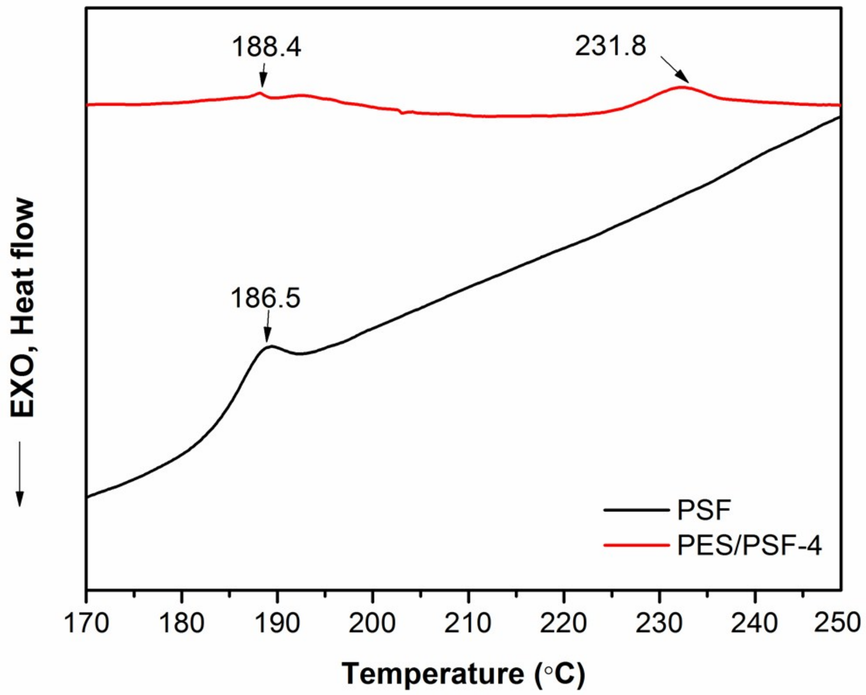

Fig. S1. Differential scanning calorimetric (DSC) curves of PSF and PES/PSF-4 membranes under N_2 atmosphere.

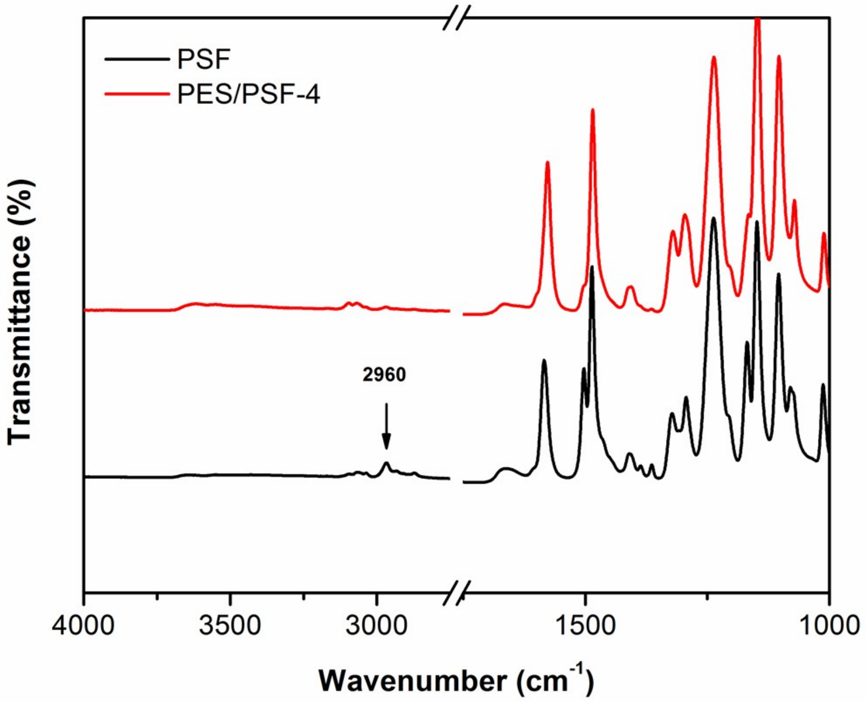

Fig. S2. FTIR spectra of the PSF membrane and PES/PSF-4 membrane.

Table S2. Pure water flux, BSA rejection and contact angle of the PES/PSF-4 membrane.

Membrane	Pure water flux ($L\cdot m^{-2}\cdot h^{-1}$)[a]	BSA rejection (%)[b]	Contact angles
PES/PSF-4	816.9 ± 25.0	43.8± 1.8	84.5±0.92

[a] Pure water flux was measured in a dead-end ultrafiltration testing system at 0.1 MPa and DI water was used as the feed solution.
[b] BSA rejection was measured in a dead-end ultrafiltration testing system at 0.1 MPa and $1 g\cdot L^{-1}$ BSA was used as the feed solution.
